# Occupational exposure limits and REACH requirements in workplace risk management: perspectives of labor inspectors and OSH specialists

**DOI:** 10.1093/annweh/wxag075

**Published:** 2026-09-10

**Authors:** Piia Taxell, Milja Koponen, Linda Schenk, Linda Paegle, Linda Matisāne, Susann Wolf, Aurora Moen, Steen Mollerup, Helge Johnsen, Davor Kontić, Jenni Kaisto, Tiina Santonen

**Affiliations:** Finnish Institute of Occupational Health, P.O. Box 40, 00032 Helsinki, Finland; Finnish Institute of Occupational Health, P.O. Box 40, 00032 Helsinki, Finland; Institute of Environmental Medicine, Karolinska Institutet, 17177 Stockholm, Sweden; Institute of Occupational Safety and Environmental Health, Riga Stradiņš University, Dzirciema iela 16, 1007 Riga, Latvia; Institute of Occupational Safety and Environmental Health, Riga Stradiņš University, Dzirciema iela 16, 1007 Riga, Latvia; National Institute of Occupational Health, Pb 5330 Majorstuen, 0304 Oslo, Norway; National Institute of Occupational Health, Pb 5330 Majorstuen, 0304 Oslo, Norway; National Institute of Occupational Health, Pb 5330 Majorstuen, 0304 Oslo, Norway; National Institute of Occupational Health, Pb 5330 Majorstuen, 0304 Oslo, Norway; Department of Environmental Sciences, Jožef Stefan Institute, Jamova 39, 1000 Ljubljana, Slovenia; Finnish Institute of Occupational Health, P.O. Box 40, 00032 Helsinki, Finland; Finnish Institute of Occupational Health, P.O. Box 40, 00032 Helsinki, Finland

**Keywords:** occupational exposure limit, REACH, workplace safety, chemical risk, labor inspector, OSH specialist

## Abstract

Occupational exposure limits (OELs) under occupational safety and health (OSH) legislation, and authorisation and restriction requirements under the chemicals regulation REACH, are the key regulatory tools for managing workplace chemical exposures in the EU. The present study examines how OELs and REACH requirements are interpreted and implemented at the workplace level, and how they impact workplace chemical safety. A mixed-methods study was conducted in Finland, Latvia, Norway, and Slovenia. An online survey targeted labor inspectors (*n* = 85), and company OSH managers (*n* = 323) in 3 sectors: chemical industry, metal industry, and motor vehicle maintenance and repair. In addition, semistructured interviews were conducted with labor inspectors (*n* = 19) and OSH experts (*n* = 30), including company and organization representatives and OSH service providers. Survey data were analyzed descriptively, and inductive content analysis was used for the interview data. Awareness of OELs and REACH requirements was reported as highly variable across workplaces in all participating countries, with larger companies higher in the supply chain demonstrating greater awareness and implementation capacity. Safety data sheets and chemical suppliers were the most frequently reported information sources. OELs were recognized as reference values for exposure and risk assessment, while REACH restrictions and authorisations were more often associated with discontinuation of substance use. Reported barriers to implementation included limited financial resources, insufficient competence in smaller companies, complex legislation, and weak supply chain communication. Positive examples included mandatory training obligations (eg diisocyanate restriction) and proactive inspection practices.

What's Important About This Paper This is the first study to compare the implementation of occupational exposure limits (OELs) and REACH authorisation and restriction requirements at workplace level. It offers valuable insights into workplace awareness, the practical impacts of these regulatory tools, and the challenges that may hinder their impact. The findings can support policymakers, labor inspectorates, and occupational health and safety professionals in promoting the effective implementation of legislation so that it more consistently translates into safer working conditions.

## Introduction

Occupational exposure limits (OELs) have a long history as a tool for regulating occupational chemical exposure (Deveau et al. 2015), and are recognized as having an important role in supporting workplace chemical risk assessment and risk management (Lißner and Zayzon 2011; Maier et al. 2015, 2025; Schenk 2020). Previous studies have linked statutory OELs with improved exposure control and reduced exposure levels (Tuomi et al. 2014; Havet et al. 2018; Havet and Penot 2022).

In the EU, OELs are set under 2 directives: the Chemical Agents Directive (CAD) (EC 1998) and the Carcinogens, Mutagens and Reprotoxic substances Directive (CMRD) (EC 2004). Member States are obliged to establish corresponding OELs in their national legislation at levels not exceeding the EU OELs. Additional national OELs may also be set.

During recent years, occupational exposure to chemicals has received increasing attention also under the EU chemicals regulation REACH (EC 2006). Several substances, whose risks are mainly related to occupational exposure, have been included in the authorisation process (eg Cr(VI) compounds, trichloroethylene) or in the restriction process (eg *N*-methyl-2-pyrrolidone [NMP] and related aprotic solvents, diisocyanates) under the REACH regulation. Certain restrictions (eg NMP) include harmonized derived no-effect levels (DNELs) for occupational exposure. These DNELs resemble health-based statutory OELs.

A significant decline in the use volumes of substances subject to REACH authorisation has been demonstrated in previous studies (Sackmann et al. 2018; ECHA 2022). A recent desktop study suggests that the authorisation process may also enhance occupational safety, as authorisation decisions often require additional control and monitoring measures beyond those proposed by the applicant (Deubner et al. 2024). However, an enforcement project evaluating companies’ adherence to authorisation requirements revealed a notably high rate of noncompliance with the required conditions of use (ECHA 2023).

While OELs, authorisations, and restrictions are acknowledged as interchangeable regulatory tools for managing occupational chemical exposure within the EU, there are currently no definitive guidelines for choosing the most suitable tool, or combination of tools, for a specific case. In addition, there is a scarcity of studies that compare the effects of OELs and REACH authorisation and restriction requirements at the workplace level. Understanding the impact of these different regulatory tools is essential for assessing the coherence and effectiveness of the regulatory framework.

The present study aims to provide insights into this issue by examining the interpretation and implementation of OELs and REACH authorisation and restriction requirements at the workplace level. The study considers the viewpoints of both labor inspectors and OSH specialists.

## Methods

The study was conducted in parallel in 4 European countries (Finland, Latvia, Norway, and Slovenia) under the EU-wide research program *Partnership for the Assessment of Risks from Chemicals* (PARC) (EC 2022), and included the following parts:

1. online survey to (i) labor inspectors inspecting workplace chemical safety (“inspector survey”), and to (ii) OSH managers in companies of 3 industry sectors; chemical industry, metal industry, and motor vehicle maintenance and repair (“workplace survey”); and
2. semistructured interviews with (i) labor inspectors specialized in chemical safety (“inspector interviews”), and with (ii) OSH experts, including representatives from employees and employer organizations and companies of the 3 target sectors, as well as OSH and occupational health service providers (“OSH expert interviews”).

The methodology was selected to enable the gathering of perspectives from a larger group of respondents via surveys, while also facilitating a more in-depth discussion of the topics through interviews. The surveys were conducted from October 2023 to April 2024, and the interviews from May 2024 to April 2025.

Ethical assessment and approval of the study were carried out at each partner institute according to the policies and laws of the institute and country in question.

### Online survey

The inspector survey targeted labor inspectors inspecting workplace chemical safety. In Finland, Latvia, and Slovenia, the survey was distributed through the national and/or regional labor inspectorates. In Norway, a distribution list was provided by the Norwegian Labor Inspection Authority. Table 1 presents the response rate and the number of responses received and included in the analysis per country.

**Table 1:** Number of survey responses and interviewees per country.

	Finland	Latvia	Norway	Slovenia	Total
**Inspector survey**					
Responses received/included	26	32	11	16	85
Response rate	30%	30%	52%	33%	…
Experience as an inspector:					
<2 yr	2	5	2	2	11 (13%)
2 to 5 yr	10	1	1	0	12 (14%)
>5 yr	14	26	8	13	61 (73%)
**Workplace survey**					
Responses received	89	65	185	10	349
Response rate	12%	30 to 45%^a^	16%	10%	…
Responses included^b^	85	62	166	10	323
Industry sector:					
Chemical industry	40	8	20	6	74 (23%)
Metal industry	20	18	87	4	129 (40%)
Motor vehicle maintenance and repair	25	10	59	0	94 (29%)
Several target sectors^c^	…	26	…	…	26 (8%)
Company size:					
<10 employees	13	6	28	…	47 (15%)
10 to 49 employees	37	10	97	…	144 (45%)
50 to 250 employees	23	32	28	6	83 (26%)
>250 employees	11	14	13	4	42 (13%)
**Interviewees**					
Inspectors	2	9	5	3	19
Company representatives	6	6	4	1	17
Organization representatives^d^	3	…	4	3	7
Other OSH experts^e^	…	…	5	1	6

^a^An approximated response rate as the survey was distributed also by external parties (see Supplementary Appendix 1 for further details). ^b^A part of the received responses was excluded from the analysis since the respondents did not belong to the target sectors. ^c^In Latvia, a part of the OSH managers is outsourced and works for several companies in different target sectors. ^d^Employees’ and employers’ organizations related to the 3 target sectors. ^e^Representatives from occupational health care and OSH consultancy services.

The workplace survey targeted OSH managers in 3 industry sectors: the chemical industry (NACE C20), the metal industry (NACE C24 & C25), and the motor vehicle maintenance and repair (NACE G45.2). These sectors were selected because they are affected by recent updates to EU OELs and REACH authorisation and restriction requirements, and cover both industrial and nonindustrial workplaces at various levels in the chemical supply chain. A detailed description of the recruitment of the workplace survey respondents is presented in Supplementary Appendix 1. Table 1 presents the response rate and the number of responses received and included in the analysis per country, industry sector, and company size.

The questionnaires for the survey were prepared in English and translated into the main national languages of the participating countries. They consisted of multiple-choice questions, some of which allowed for further specification of responses with open text. To ensure comprehensibility, the draft questionnaire for the workplace survey was reviewed by an OSH expert not specialized in chemicals legislation.

The inspector survey covered the following topics:

- years of experience as a labor inspector;
- observed general awareness of workplaces on OELs and REACH authorisation and restriction requirements;
- observed measures triggered at workplaces by OELs and REACH authorisation and restriction requirements;
- identified challenges in the implementation of OELs and REACH authorisation and restriction requirements;
- perceived support for the work of labor inspectors from OELs and REACH authorisation and restriction requirements.

The workplace survey covered the following topics:

- industry sector and company size;
- workplace chemical risk assessment;
- following legislative updates and related information sources;
- measures triggered by OELs and REACH authorisation and restriction requirements;
- identified challenges in the implementation of OELs and REACH authorisation and restriction requirements.

The surveys were conducted anonymously using the Webropol survey tool (https://webropol.com/). Statistical Package for the Social Sciences (SPSS) software was used to analyze the survey data.

### Semistructured interviews

The interviews targeted labor inspectors specialized in chemical safety, and OSH and chemical safety experts, including experts from employer and employee organizations and companies in the 3 target sectors. Knowledge on the implementation of OELs and REACH requirements at the workplace level was expected from the interviewees. A detailed description of the recruitment of the interviewees is presented in Supplementary Appendix 2. Table 1 presents the number of interviewees per country and target group.

The interview guidelines, which included open-ended interview questions and instructions for interviewers, were prepared in English and translated into the primary national languages of the participating countries. The following topics were covered in the interviews:

- awareness of workplaces on OELs and REACH authorisation and restriction requirements;
- aspects influencing and ways to improve the awareness;
- impact of OELs and REACH authorisation and restriction requirements on workplace safety and their consideration in workplace risk assessment;
- identified challenges and good practices related to workplace implementation of OELs and REACH authorisation and restriction requirements;
- use of exposure measurements and limit values.

In the inspector interviews, support for the work of labor inspectors from OELs and REACH authorisation and restriction requirements was additionally discussed.

Interviews were organized as online (group) interviews (MS Teams), with separate interviews for the inspectors and the OSH experts (1 to 9 persons per interview). The interviews were conducted in the primary national language of each participating country, lasted for 1 to 2 h, and were recorded and transcribed in MS Teams.

The interviewees received the interview topics and questions approximately 1 wk in advance, along with a letter describing the study's aims, data security details, personal data processing, and voluntariness. The interview questions were displayed on-screen during the interview. Before starting the interview, the interviewees either signed their informed consent to participate in the study or provided it orally while being recorded. Interviews were conducted and moderated by experienced researchers of each partner institute according to written guidelines.

All identifiable recordings and transcriptions were stored in accordance with the partners’ data protection and data security guidelines. Pseudonymized data was shared between the partners in an MS SharePoint workspace provided by the PARC program.

The analysis of the interview transcriptions began with pseudonymization of the material, ie all names of individuals or companies were replaced with identifiers, formed from country prefix (FI, LV, NO, or SI), letter E for experts and I for inspectors, and a running number. As prior research on the topics of this study is limited, an inductive content analysis of the interview data was conducted. Transcriptions were read repeatedly to identify codes and categories of different topics, searching for repeating topics or keywords (Patton 2002; Elo and Kyngäs 2008).

The initial coding was performed manually in the original language by the researchers conducting the interviews, and was later discussed with the larger project group to ensure a common understanding. The codes were arranged by categories, counted, and the number of appearances of each topic was marked in the documents. Descriptive quotes, 1 to 2 per code, were selected to show support for the selected codes, and the codes and quotes were translated into English.

The codes were compared between the participating countries to identify similarities and differences. The combined analysis was led by the Finnish Institute of Occupational Health and discussed repeatedly in the larger project group to ensure that country-specific details were understood correctly. The final set of quotes was selected in agreement between all authors.

## Results

In the following, the findings from the surveys and interviews are presented in 5 topics: (i) workplace awareness of legislation, (ii) ways to improve the awareness, (iii) impact of legislation, (iv) hindrances of impact, and (v) use of exposure measurements and limit values. For each topic, the results from the 4 data streams—inspector survey, workplace survey, inspector interviews, and OSH expert interviews—are presented separately.

### Awareness of legislation

#### Inspector survey

**Figure 1:**
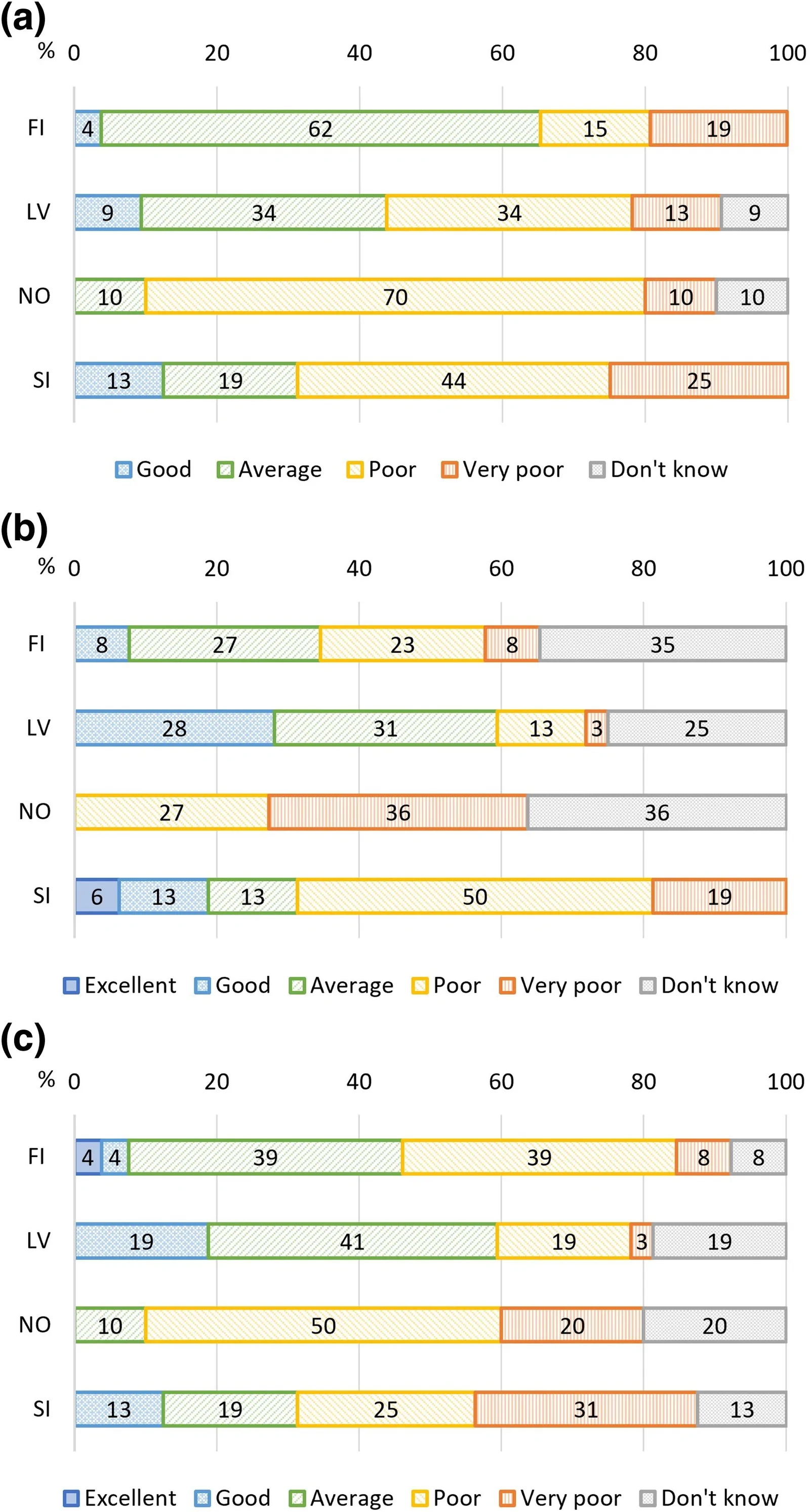
General awareness of workplaces on a) occupational exposure limits (*n* = 84), b) REACH authorisation requirements (*n* = 85), and c) REACH restriction requirements (*n* = 84), according to inspector survey responses from Finland (FI), Latvia (LV), Norway (NO), and Slovenia (SI).

General awareness of workplaces on OELs was rated as “average” to “poor” by the majority of the respondents in all 4 countries (Fig. 1). A similar pattern was observed for the awareness of the REACH authorisation and restriction requirements, except for Norway, where awareness of authorisation requirements was rated as “poor” to “very poor.” Notably, a significant fraction of the inspectors responded not knowing the level of awareness of workplaces, especially regarding the REACH authorisation requirements.

#### Workplace survey

Although the workplace survey did not specifically address awareness, a question on compliance with the legislation revealed that a relatively high share of responding companies had not yet considered their compliance with OELs (31%), REACH authorisation requirements (29%), or REACH restriction requirements (21%) (Fig. S1).

**Figure 2:**
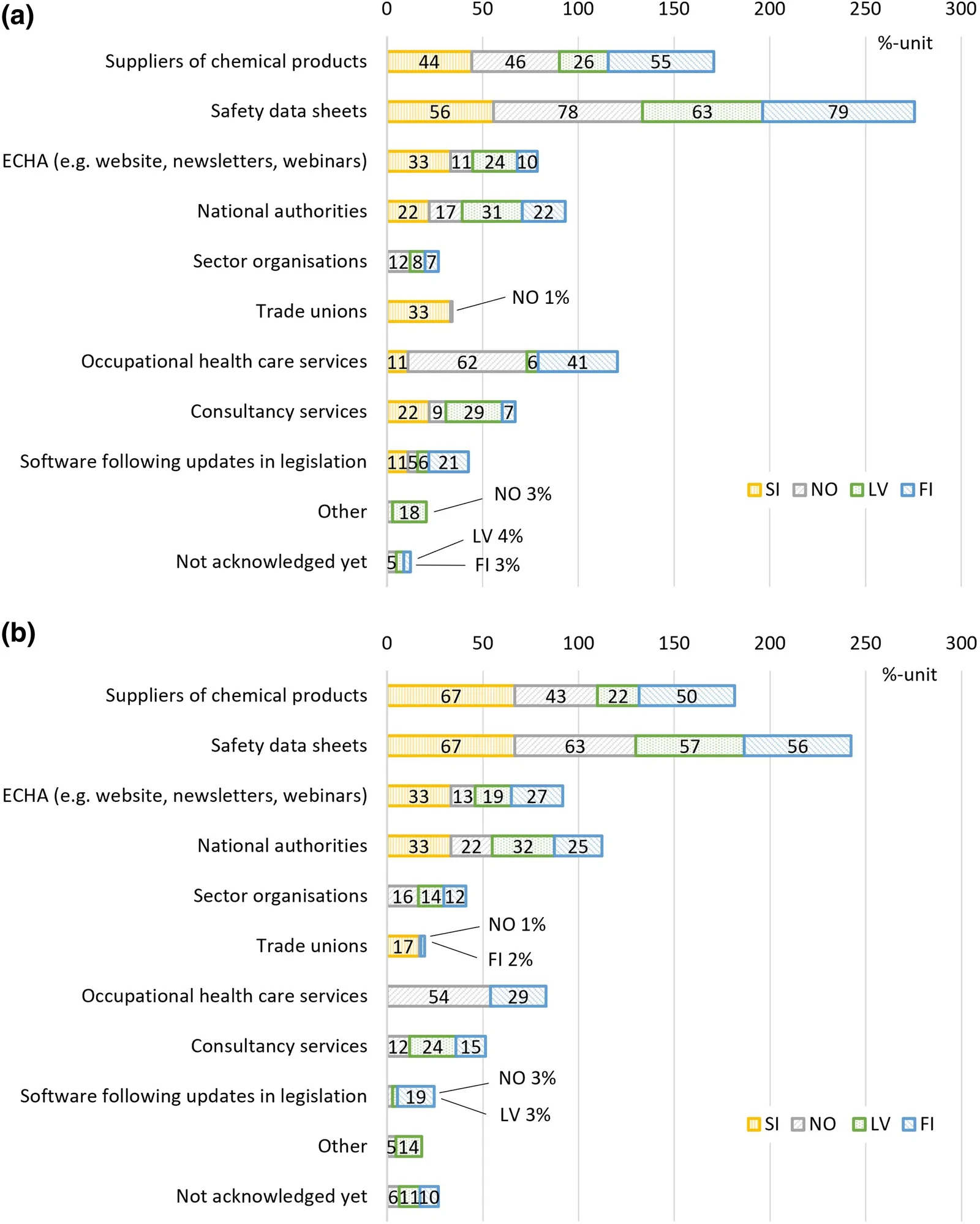
The most important information sources on a) updates in occupational exposure limits (*n* = 258), and b) REACH authorisation and restriction requirements (*n* = 206), according to workplace survey responses from Finland (FI), Latvia (LV), Norway (NO), and Slovenia (SI). The bars correspond to the percentage of respondents in each country that selected the option mentioned. The respondents were allowed to select 1 to 3 options.

Safety data sheets and chemical suppliers were identified as the most important information sources on both OELs and the REACH requirements by the workplace survey respondents in all countries (Fig. 2). In Norway and Finland, where occupational health care has a statutory role in assessing health risks and providing related guidance to workplaces, it was identified as an important additional information provider, especially on OELs.

#### Inspector interviews

The inspectors from all participating countries reported that the level of awareness of OELs and REACH requirements at workplaces is highly variable (Table S1). Company size was mentioned as a major factor influencing awareness levels, with larger companies having more resources and higher awareness compared to smaller ones. The industry sector and position in the supply chain were also stated to have an impact.

The level of awareness, and chemical safety in general, was also mentioned to be highly dependent on the resources the companies have for using outsourced expertise, and the quality of these services.

Especially in Norway, OELs seem to be widely recognized, whereas REACH authorisation and restriction requirements are not as familiar.

#### OSH expert interviews

In line with the inspector interviews, OSH experts in all 4 countries reported a wide variation in the level of awareness across companies (Table S1). In general, larger companies of the chemical industry were considered to have the best knowledge of both OELs and REACH requirements. REACH requirements, in particular, were mentioned to be less well known outside the chemical sector. Also, companies active in sector associations were reported to be more aware of the legislation. Finnish and Norwegian OSH experts also mentioned the importance of good-quality occupational health care services in promoting awareness.

### Ways to improve awareness

#### Inspector interviews

Training of OSH experts, in-house or outsourced, was mentioned by the Latvian and Slovenian inspectors as a way to increase the level of awareness at workplaces (Table S1). Norwegian and Finnish inspectors had experienced that the diisocyanate restriction, requiring mandatory training of employees and their supervisors, was an efficient way to raise awareness.

Campaigns and campaign materials were seen as a possible solution for raising awareness by the Latvian and Norwegian inspectors. It was stated, however, that it would also depend on the companies to utilize these efforts.

Latvian inspectors noticed that more targeted inspection activities of chemical risks could potentially increase awareness and, thereby, the level of implementation of legislation. In Finland, it is a common practice to focus the inspection activities on new or updated OELs when they are introduced.

#### OSH expert interviews

Company management's commitment to occupational and chemical safety was seen as a prerequisite for increasing awareness by the Finnish, Norwegian, and Slovenian OSH experts (Table S1). The Finnish and Latvian experts mentioned the utilization of legal compliance services as a practical tool for increasing awareness. Also, well-defined practices for purchasing chemicals were specifically mentioned by the Finnish experts.

Regular training of personnel working with chemicals was identified as essential for keeping the knowledge up-to-date. One Finnish OSH expert stated that more training on chemical safety should be included already in vocational education.

The OSH experts from Finland, Latvia, and Norway called for a more consultative attitude and guidance from the labor inspectors. Strengthening the national labor inspectorate and its ability to assist businesses across various sectors was deemed necessary, particularly by the Norwegian and Latvian interviewees.

### Impact of legislation

#### Inspector survey

Most respondents in all 4 countries considered that both updating/setting of new OELs and the introduction of new REACH authorisation or restriction requirements have only “sometimes” or “seldom” triggered measures at workplaces (Fig. S2). However, a significant fraction of the respondents indicated that they do not know if measures are triggered, especially by REACH authorisation requirements.

**Figure 3:**
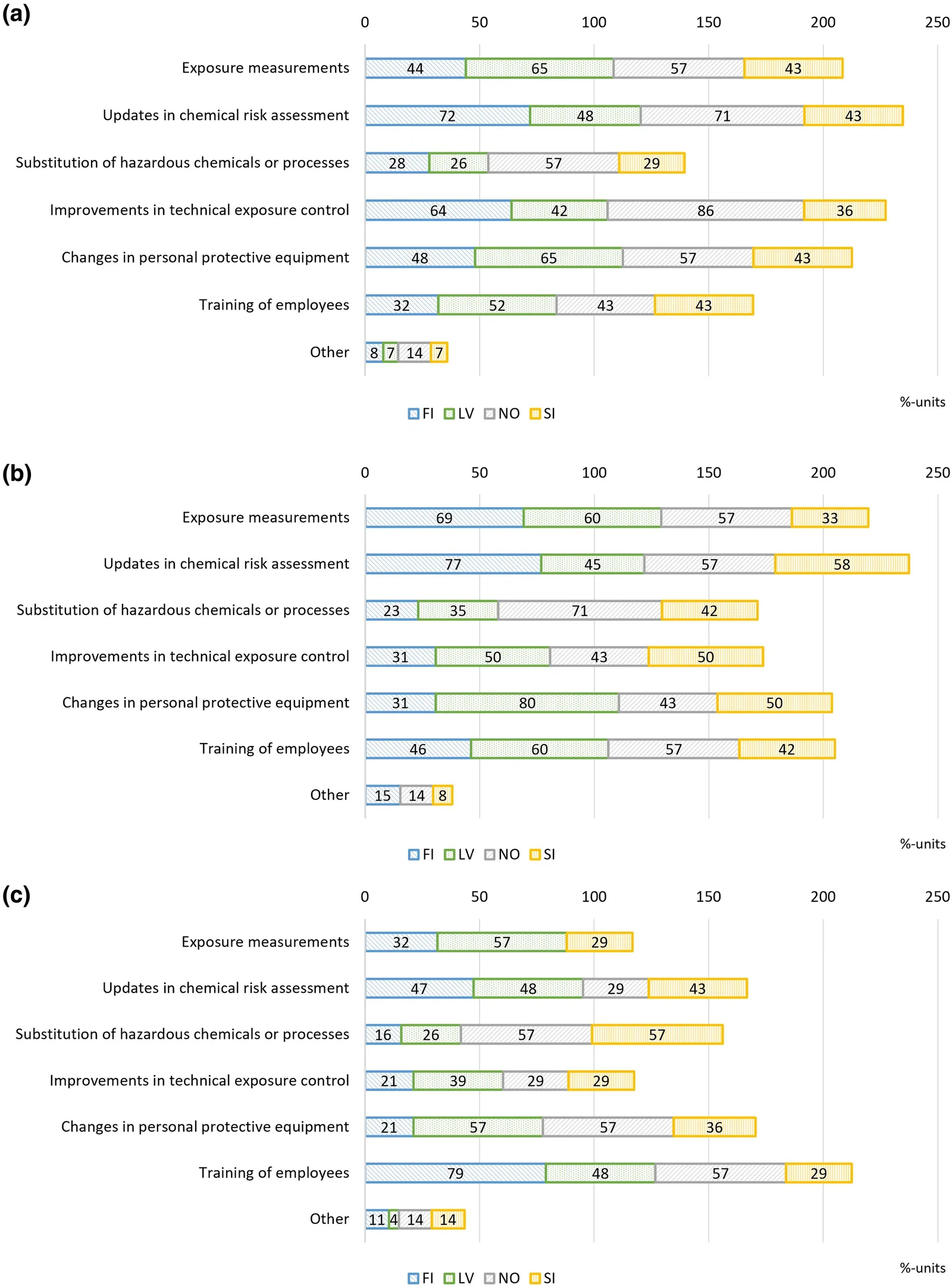
Measures triggered at workplaces by a) setting or updating of occupational exposure limits (*n* = 77), by b) REACH authorisation requirements (*n* = 52), and by c) REACH restriction requirements (*n* = 63), according to inspector survey responses from Finland (FI), Latvia (LV), Norway (NO), and Slovenia (SI). The bars correspond to the percentage of respondents in each country that selected the option mentioned. The respondents were allowed to select several options.

Among the inspectors who had reported measures to be triggered, a wide variety of measures were identified (Fig. 3). Both OEL updates and REACH authorisations were identified as triggers for updates in workplace chemical risk assessment and exposure measurements. OEL updates were often mentioned to trigger improvements in technical control measures, and both OEL updates and REACH requirements changes in personal protective equipment. Training of employees was the most often reported measure for REACH restrictions, probably reflecting the training requirements under the diisocyanate restriction.

OELs were considered to support the work of labor inspectors by 67% of the respondents (figure not included). In the open responses, the most often reported reasoning was that OELs provide an objective reference point for evaluating the adequacy of exposure control measures and enforcing related obligations in a workplace (all countries). OELs were also mentioned to help inspectors demand exposure measurements (FI, NO) and to support communication with workplaces (FI).

The inspectors who did not consider OELs to be of support, commented that they themselves or their colleague inspectors are not familiar with the OELs (FI, LV), or that there is no possibility to consider OELs during an inspection due to different focus, limited time, or lack of even a basic risk assessment at the workplace (FI, NO, SI).

Support from REACH requirements was somewhat less well recognized; 32% and 40% of the respondents considered REACH authorisations and restrictions, respectively, to support inspection work (figure not included). Clear obligations and justifications for requiring actions were mentioned as their main strengths (FI, NO, SI), provided that inspectors receive training in inspecting compliance with REACH requirements (NO). Identified challenges included a lack of knowledge of REACH requirements among the inspectors (FI, LV, NO).

#### Workplace survey

**Figure 4:**
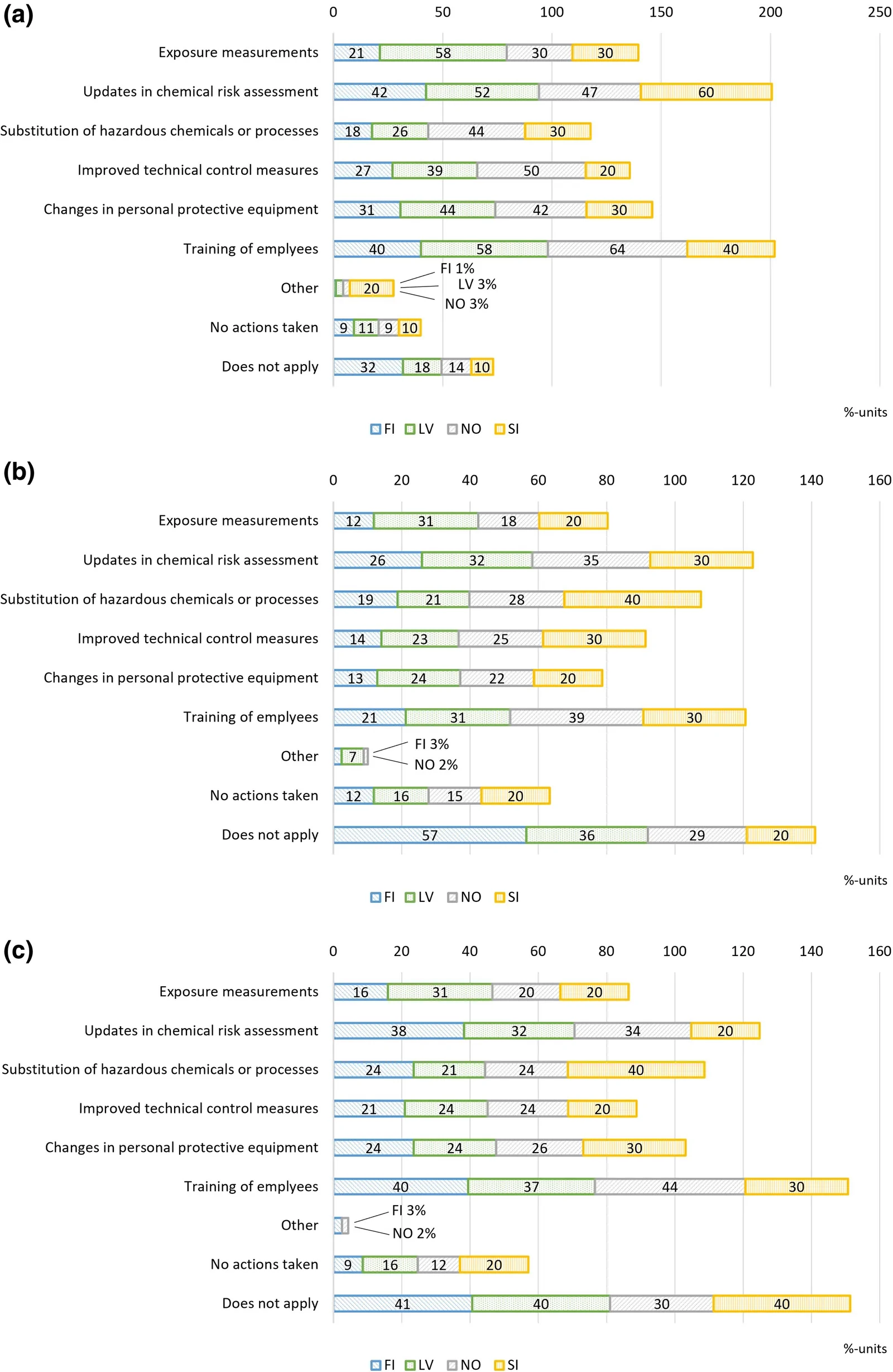
Measures triggered at workplaces by a) setting or updating of occupational exposure limits (*n* = 320), by b) REACH authorisation requirements (*n* = 319), and by c) REACH restriction requirements (*n* = 314), according to workplace survey responses from Finland (FI), Latvia (LV), Norway (NO), and Slovenia (SI). The bars correspond to the percentage of respondents in each country that selected the option mentioned. The respondents were allowed to select several options.

A wide variety of measures triggered by updates in OELs and REACH requirements were reported in the workplace survey (Fig. 4). Updates in workplace chemical risk assessment and employee training were the most frequently reported measures, both for OEL updates and REACH requirements. Substitution of hazardous chemicals or processes was the third most prominent measure reported for REACH authorisation and restriction requirements, and changes in personal protective equipment for OEL updates.

#### Inspector interviews

In Finland and Norway, the inspectors considered OELs important for interpreting exposure measurement data and assessing risks (Table S2). REACH authorisations, at least in some cases, had been effective in causing process changes and substitution of the most hazardous chemicals. Mandatory training, as introduced by the diisocyanate restriction, was specifically welcomed by the Norwegian inspectors.

OELs were also considered to support inspectors in giving compelling orders to workplaces to conduct exposure measurements and to implement risk management measures. Direct support to inspectors from the REACH requirements was less well recognized.

#### OSH expert interviews

Finnish and Norwegian OSH experts considered OELs to have an important role in the everyday OSH work, whereas REACH authorisations and restrictions were seen as powerful regulatory tools for discontinuing the use of a substance (Table S2). The Finnish OSH experts also raised concerns about whether REACH requirements were too stringent in some cases, hampering European businesses.

### Hindrances of impact

#### Inspector survey

The Finnish and Norwegian labor inspectors, and to a lesser extent the Slovenian inspectors, had identified challenges that hinder the implementation of OELs and REACH requirements at the workplace level (Fig. S3). Latvian inspectors had identified challenges related to OELs only.

Lack of knowledge of OELs (FI, LV, NO) and on REACH authorisation and restriction requirements (FI, NO), especially in the smaller companies, was the most often mentioned challenge in the open responses. Related to OELs, additional challenges mentioned included lack of resources (eg to perform exposure measurements) (FI, LV), lack of knowledge on chemical exposures and chemical safety in general (FI, NO), lack of interest in OELs and related issues (FI, LV), and technical challenges (FI, NO, SI).

The REACH authorisation process was considered too resource-demanding, especially for smaller companies (FI). For REACH restrictions, the lack of resources in companies (FI), products imported from outside the EU that do not meet the restriction requirements (SI), and the lack of high-quality diisocyanate training (FI) were mentioned as specific challenges.

#### Workplace survey

Only 7% of respondents reported challenges related to compliance with OELs or REACH restrictions, and 5% with REACH authorisations, at their workplace (Fig. S1).

For OELs, the most frequently mentioned challenges in the open responses included technical difficulties in reaching the OELs (all countries) and missing information on OELs in general (LV, NO). For REACH requirements, the lack of information and general knowledge on the issue (FI, LV, NO), as well as increased costs (FI, NO, SI), were the most often mentioned challenges.

#### Inspector interviews

Lack of competence, especially in smaller companies, was reported by the inspectors in all participating countries as a hindrance that prevents companies from acting in compliance with the legislation (Table S2). Insufficient basic level of chemical safety at the workplaces is often a hindrance and may also prevent inspectors from addressing OELs or REACH requirements during the inspection. Finnish inspectors also mentioned the lack of financial resources as an important hindrance.

The Latvian and Norwegian inspectors mentioned the complexity of the legislation as a potential hindrance that may prevent the objectives of the legislation from being realized. Also, insufficient supply chain communication was considered a challenge by the Norwegian inspectors.

#### OSH expert interviews

Lack of financial resources was recognized by the OSH experts in all 4 countries as a major barrier preventing companies from achieving compliance (Table S2). Technical solutions to manage risks are often expensive, and proving their necessity to company management can be difficult. Also, the lack of expertise in companies was recognized as a challenge by the Norwegian and Slovenian OSH experts.

A practical challenge raised by the Finnish and Norwegian OSH experts was that OELs are becoming increasingly stringent, making them difficult to reach with technical measures. Also, the use of respiratory protection may be challenging in specific work environments. Norwegian OSH experts pointed out that it may take a long time to plan and implement all the necessary measures. The complexity of the legislation was mentioned as a hindrance by the Finnish OSH experts; chemicals legislation was considered challenging to follow and interpret, even for the most experienced experts.

Challenges related to the general safety attitude were raised in discussion in Slovenia. A Finnish OSH expert also pointed out that the legislation of restrictive business practices may hinder companies from closely collaborating on OSH issues. Finnish and Norwegian OSH experts commented on chemical inventory and risk assessment software, which usually do not consider OELs or REACH.

### Use of exposure measurements and limit values

#### Workplace survey

**Figure 5:**
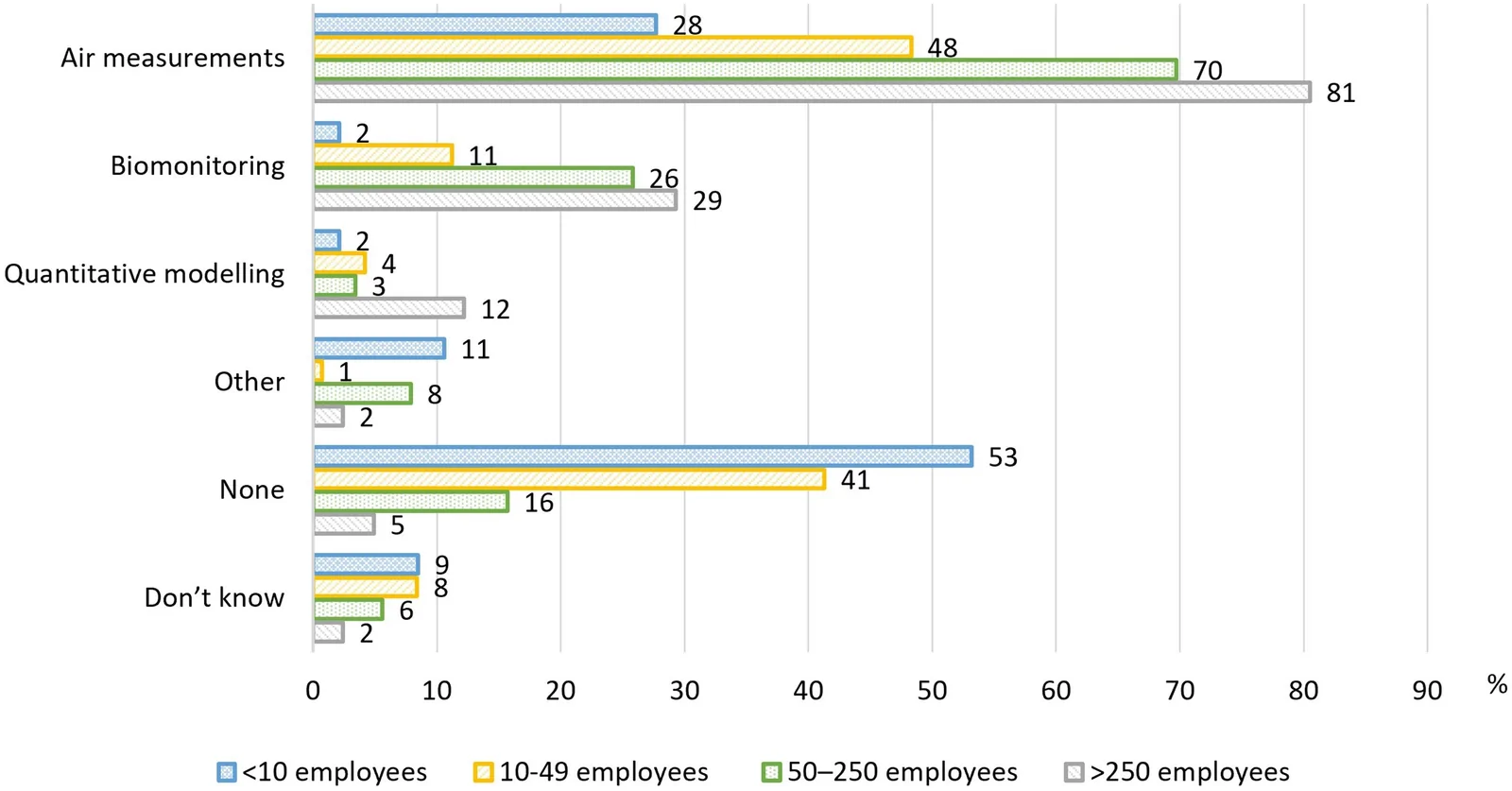
Use of exposure measurements or quantitative modelling in the assessment of employees’ exposure to chemical agents according to workplace survey responses, grouped by company size (all countries combined; *n* = 321). The respondents were allowed to select several options.

Occupational hygiene measurements were carried out in 55% of the responding companies, with the rate correlating with company size (Fig. 5). Also, the rate of using biomonitoring for exposure assessment increased with company size.

#### Inspector interviews

The practices inspectors reported for demanding exposure measurements varied across the 4 countries (Table S3): In Norway, measurements may be demanded when there have been significant changes in production or if a specific amount of time has passed since the last measurement. In Finland and Latvia, inspectors may require measurements when, eg, dust or smoke can be seen or smelled, occupational diseases have been diagnosed, or employees complain of symptoms. In Slovenia, inspectors seldom require measurements.

Finnish and Latvian inspectors considered exposure levels as acceptable as long as measurement results did not exceed the limit values. In Norway, measurement standards (eg EN 689) are, in principle, recommended to be followed to address measurement uncertainties, but the Norwegian inspectors mentioned that, in practice, it is quite common to accept compliance when individual measurement results remain just below the limit values.

#### OSH expert interviews

Many of the Finnish, Latvian and Norwegian OSH experts mentioned that measurements take place regularly, following a pre-established plan (Table S3). Also, updates in processes or OELs, recommendations from occupational health care services, employee complaints, and requirements from authorities were reported to trigger measurements. In Slovenia, medical examinations were mentioned to have taken over the role of exposure measurements.

OELs were acknowledged as the primary reference value for exposure measurements by the Finnish, Latvian, and Norwegian OSH experts. REACH-derived DNELs were not recognized to have a similar role. However, some of the interviewees in Finland and Norway reported consistently using the most stringent of the limit values.

Finnish, Latvian, and Norwegian OSH experts reported that measurement standard EN 689, or related companies’ internal guidelines, were followed to address uncertainties in exposure measurements. Exposure levels just below the limit values were considered adequate by the Slovenian OSH experts.

## Discussion

According to the inspector and OSH expert interviews, awareness of legislative updates varies widely among companies, being higher in larger companies and those further up the supply chain. This suggests that new legislative requirements, either OELs or REACH requirements, may have a more significant impact on larger companies that already maintain a higher baseline level of chemical safety, and emphasizes the importance of enhancing awareness, especially among smaller companies, to ensure the intended outcome of the legislation. Impact of company size, sector and available resources on legislative compliance and level of workplace safety has also been identified in earlier studies (Schenk 2013; Schenk and Antonsson 2015; Abderhalden-Zellweger et al. 2021; Edwin et al. 2021; Fransman et al. 2023).

According to the workplace survey, safety data sheets and chemical suppliers are the most important information sources for companies on both OEL updates and on REACH authorisation and restriction requirements. Earlier, the REF-9 project on the enforcement of compliance with REACH authorisation obligations identified low quality and quantity of supply chain communication as a major impediment (ECHA 2023). These findings underline the need to promote the quality and availability of safety data sheets and improve information flow in the supply chain as the primary means to ensure that information on legislative updates reaches all workplaces. This is particularly important for the smaller downstream user companies, whose competence and resources to follow legislative updates are limited.

The diisocyanate restriction was mentioned in the inspector interviews as an example of a legislative update on which information sharing in the supply chains has been more successful than usual. For this restriction, the suppliers have a specific role in providing training for their customers (EC 2020), which according to the inspectors improve the information flow and awareness in the customer companies. It would be useful to investigate the effects of this restriction in more detail to determine if the mandatory training requirements lead to an improved level of chemical safety, and, for example, a reduction in the number of diisocyanate-related occupational diseases.

Since the complexity of the legislation was identified as a potential hindrance to its impact, prioritizing clarity and simplicity of the legislation and related guidance should be considered when establishing new regulatory measures. In line with our findings, regulations’ clarity was found as an essential factor in the recent review addressing the influence of OSH legislation on workplace conditions in Europe (Ståhl et al. 2025).

In all the participating countries, labor inspectorates are responsible for the enforcement of OELs, and in all the countries except Slovenia, also for workplace-level enforcement of REACH authorisation and restriction requirements. However, according to the inspector survey, not all the inspectors have adequate knowledge of OELs, let alone the REACH requirements. Therefore, further training of the inspectors on these issues would be necessary for them to provide better support for the workplaces.

The inspector survey and interviews also brought up that the inspection process and limited time to conduct an inspection may hamper the discussion of OELs or REACH requirements during the inspection. Also, as even a basic workplace risk assessment is often missing, the inspections were mentioned to focus on this instead of the “next steps,” including OELs or REACH. Here, the inspection process might benefit from a stepwise approach where the same workplace would be visited several times, first focusing on the basics and gradually increasing the scope.

The role of OELs as reference values in exposure and risk assessment was underlined in the inspector and OSH expert interviews. REACH restriction or authorisation related harmonized DNELs were considered not to have a similar impact on workplace safety as OELs. Instead, REACH authorisations and restrictions were reported to often lead to the cessation of use of the substance. This is an important finding and supports the selection of the most appropriate regulatory tool for a specific case, depending on the desired outcome. According to inspector and workplace survey responses, however, any updates in legislation are likely to draw attention to the chemical in question, causing companies, eg, to update their risk assessments.

For limit values, either OELs or DNELs, to directly impact the risk assessment and risk management at a workplace, exposure measurements are needed. According to the workplace survey, exposure measurements were conducted relatively rarely in small to medium-sized companies. This may reflect not only the general level of awareness but also the limited financial resources and knowledge to conduct exposure measurements. This finding again highlights that OELs and related regulatory measures may have more impact on larger companies with a higher baseline level of OSH. On the other hand, the inspectors saw the availability of OELs as a support for demanding exposure measurements. The importance of exposure measurements is emphasized by earlier studies showing poor correlation of exposure and risk estimates with the actual levels without measurements (Coşkun Beyan et al. 2023; Schenk et al. 2025a).

For interpreting the measurement results, the interviewees indicated that in the usually larger companies where regular measurements are voluntarily made, measurement standards or related guidelines are applied to acknowledge uncertainties related to day-to-day and between-worker variation in the exposure levels, whereas in the usually smaller companies, where inspectors must enforce the measurements, individual measurement results just below the limit values are accepted. This again highlights the varying impact of the legislation depending on company size and level of expertise. In the recent SafeChrom study in Sweden, OELs were found to be actively used as reference levels in the participant companies, but the variability in exposure levels was largely overlooked (Schenk et al. 2025b).

When evaluating the results of this study, it should be noted that the workplace survey and the OSH expert interviews focused on 3 industry sectors: the chemical industry, the metal industry, and the motor vehicle maintenance and repair industry. Therefore, their findings may not be directly transferable to other industries. Companies responding to the workplace survey may also have a higher-than-average interest in chemical safety. The inspector survey and interviews, on the other hand, covered inspectors’ experience across all sectors and workplaces. Indeed, labor inspectors may be more focused on companies with less knowledge of chemical safety, which may be reflected in their responses. However, the low response rate, particularly in the workplace survey (10 to 30%), but also in the inspector survey (30% to 52%), may limit the generalizability of the findings. Inspectors and OSH managers who chose to participate are likely to be more engaged or more aware of the topic.

Despite using a harmonized survey and interview guide, differences in national labor inspection and OSH practices, along with language-related deviations in interpreting survey and interview questions and responses, could affect the comparability of the results across countries. There was also some variation in the distribution of industry sectors and company sizes in the workplace survey, as well as in the representation of target groups in the OSH expert interviews between the countries, which may impact the results. Our findings should thus be seen as a nonexhaustive range of perspectives on the role of OELs and REACH requirements in workplace risk management.

## Conclusions

Our findings show that OELs and REACH authorisation and restriction requirements may have a greater impact on larger companies, which tend to have better awareness of legislative changes and resources to conduct exposure measurements and implement risk management measures. In general, OELs are likely to support exposure and risk assessment, as well as regulating chemical exposures to specific levels. REACH authorisations and restrictions are useful regulatory tools when the goal is to discontinue the use of a substance.

Improving the quality of safety data sheets and enhancing supply chain communication should be a priority, as these are the key information sources on OELs and REACH requirements for workplaces. This is especially important for smaller companies. Mandatory training requirements, such as those under the diisocyanate restriction, appear to provide one way of facilitating information sharing in supply chains.

Labor inspectors are recommended to adopt a consultative approach to enhance awareness and effective implementation of legislation. There is a need to update and strengthen the inspection practices and expertise of inspectors to enable this.

## Supplementary Material

wxag075_Supplementary_Data

## Data Availability

The online survey data and coded interview data will be shared for research purposes on reasonable request to the corresponding author.
