# Supplementary material for: Occupational exposure limits and REACH requirements in workplace risk management: perspectives of labor inspectors and OSH specialists
Source: Ann Work Expo Health. 2026 Sep 10;70(7):wxag075. doi: 10.1093/annweh/wxag075 (PMC13559728; doi:10.1093/annweh/wxag075)
Supplement: Supplementary file 1 [file wxag075_supplementary_data.pdf]

Occupational exposure limits and REACH requirements in workplace risk management: perspectives of labour inspectors and OSH specialists

Piia Taxell [a,*], Milja Koponen [a], Linda Schenk [b], Linda Paegle [c], Linda Matisāne [c], Susann Wolf [d], Aurora Moen [d], Steen Mollerup [d], Helge Johnsen [d], Davor Kontic [e], Jenni Kaisto [a], Tiina Santonen [a]

[a] *Finnish Institute of Occupational Health, Helsinki, Finland*

[b] *Institute of Environmental Medicine, Karolinska Institutet, Stockholm, Sweden*

[c] *Institute of Occupational Safety and Environmental Health, Riga Stradiņš University, Riga, Latvia*

[d] *National Institute of Occupational Health, Oslo, Norway*

[e] *Jožef Stefan Institute, Ljubljana, Slovenia*

* Corresponding author: piia.taxell@ttl.fi; +358 30 474 2234

Appendix 1. Recruitment of workplace survey respondents

In Finland, the national register of OSH personnel (Työsuojeluhenkilörekisteri) provided email addresses of OSH managers of private companies in the three target sectors (searched by the NACE codes). The total number of email addresses received was 719, and the total number of responses received for the survey was 89 (response rate 12%). Four responses were discarded from further analysis as the respondents did not represent the target sectors.

In Latvia, the contact database of the Institute of Occupational Safety and Environmental Health at Rīga Stradiņš University was used to reach the OSH managers of the selected sectors. A total of 144 OSH managers were invited to respond to the survey. Additionally, an invitation to distribute the survey was sent to two target sector associations: the Association of Mechanical Engineering and Metalworking Industries of Latvia and the Latvian Association of Life Sciences Industry. In total, 65 responses were received, with an estimated response rate of 30–45%. Three responses were excluded as they did not belong to the target sectors.

In Norway, the Norwegian Labour Inspection Authority provided a list of companies in the three target sectors (searched by the NACE codes). Email addresses of the companies were collected from online sources by the authors. The survey was sent to OSH managers of 1180 companies, and the total number of responses received was 185 (response rate 16%). 19 responses were discarded as the respondents did not represent the target sectors.

In Slovenia, the survey was sent to 57 companies in the three target sectors through contact details provided by the Labour Inspectorate of the Republic of Slovenia (n=32) and retrieved from online sources (n=25). The Institute for Occupational Safety and the Ministry of Labour, Family, Social Affairs and Equal Opportunities distributed the survey to 40 additional companies. The total number of responses received was 10 (response rate 10%).

Appendix 2. Recruitment of interviewees

In Finland, the company and organisation representatives were recruited through the Working Committees of the three target sectors under the Centre for Occupational Safety, and through the professional networks of the authors. The Working Committees are voluntary bodies where both employee and employer organisations and larger companies are represented. Contact information for labour inspectors experienced in chemical safety was received from the national labour inspectorate.

In Latvia, OSH experts were invited to participate in interviews through an announcement distributed on the national working life portal *stradavesels.lv*. Information about the planned interviews for inspectors was communicated to the head of the Competence Development Department at the State Labour Inspectorate, who internally invited the inspectors to participate.

In Norway, representatives from companies and organizations, as well as other OSH experts, including those from occupational health services, were recruited through the professional networks of the authors. The National Labour Inspection Authority provided contact information for labour inspectors with expertise in chemical safety. An interview request was sent to selected inspectors, who subsequently distributed it internally.

In Slovenia, participants representing companies, organizations, and OSH and occupational health care professionals were identified through the authors' professional networks, assistance from the Labour Inspectorate, public databases provided by various governmental bodies, and referrals gathered during the interviews. The Labour Inspectorate and the Chamber of Commerce contributed contact details for inspectors with relevant expertise in chemical safety.

Figure S1. Challenges experienced by the companies in the implementation of occupational exposure limit values (n=256), REACH authorisation requirements (n=197), or REACH restriction requirements (n=205), according to workplace survey responses (all countries combined).

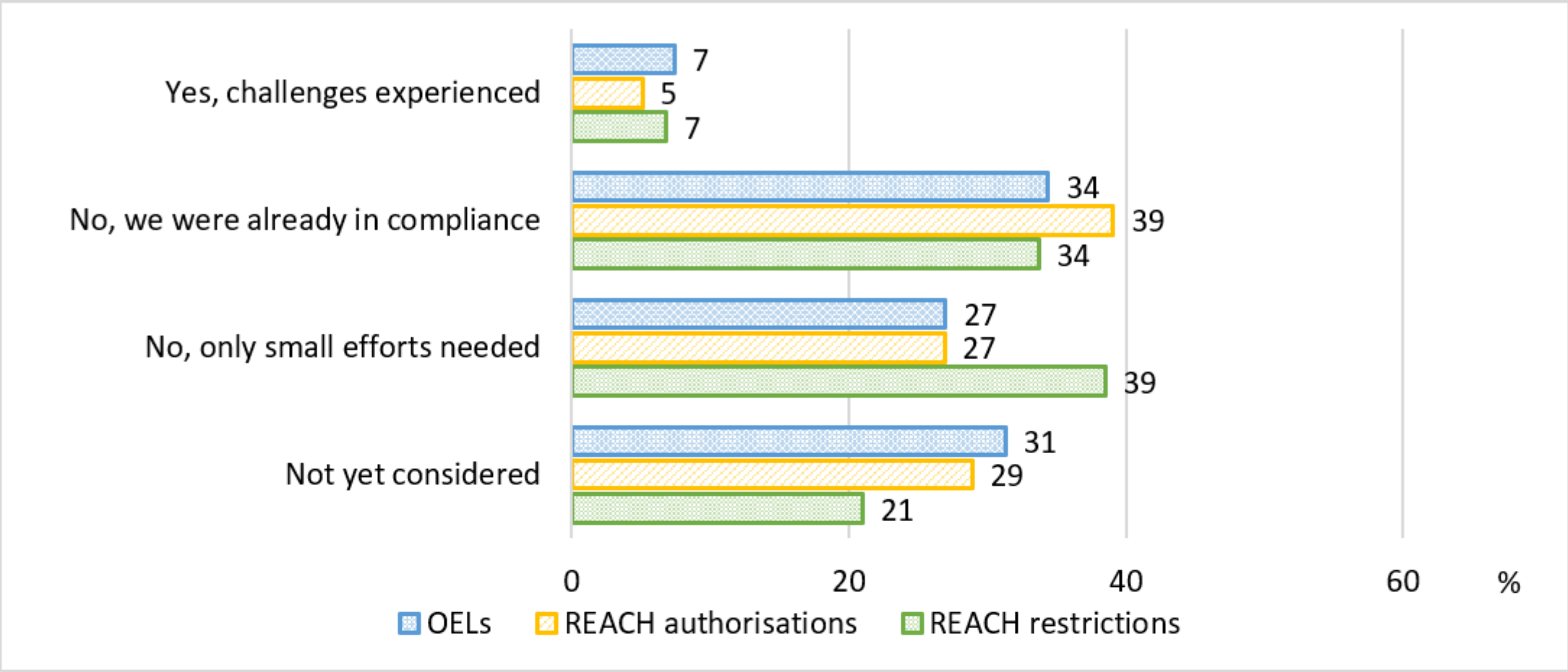

Figure S2. Frequency of measures triggered at workplaces by a) setting or updating of OELs (n=85), b) REACH authorisation requirements (n=85), and c) REACH restriction requirements (n=84), according to the inspector survey responses from Finland (FI), Latvia (LV), Norway (NO) and Slovenia (SI).

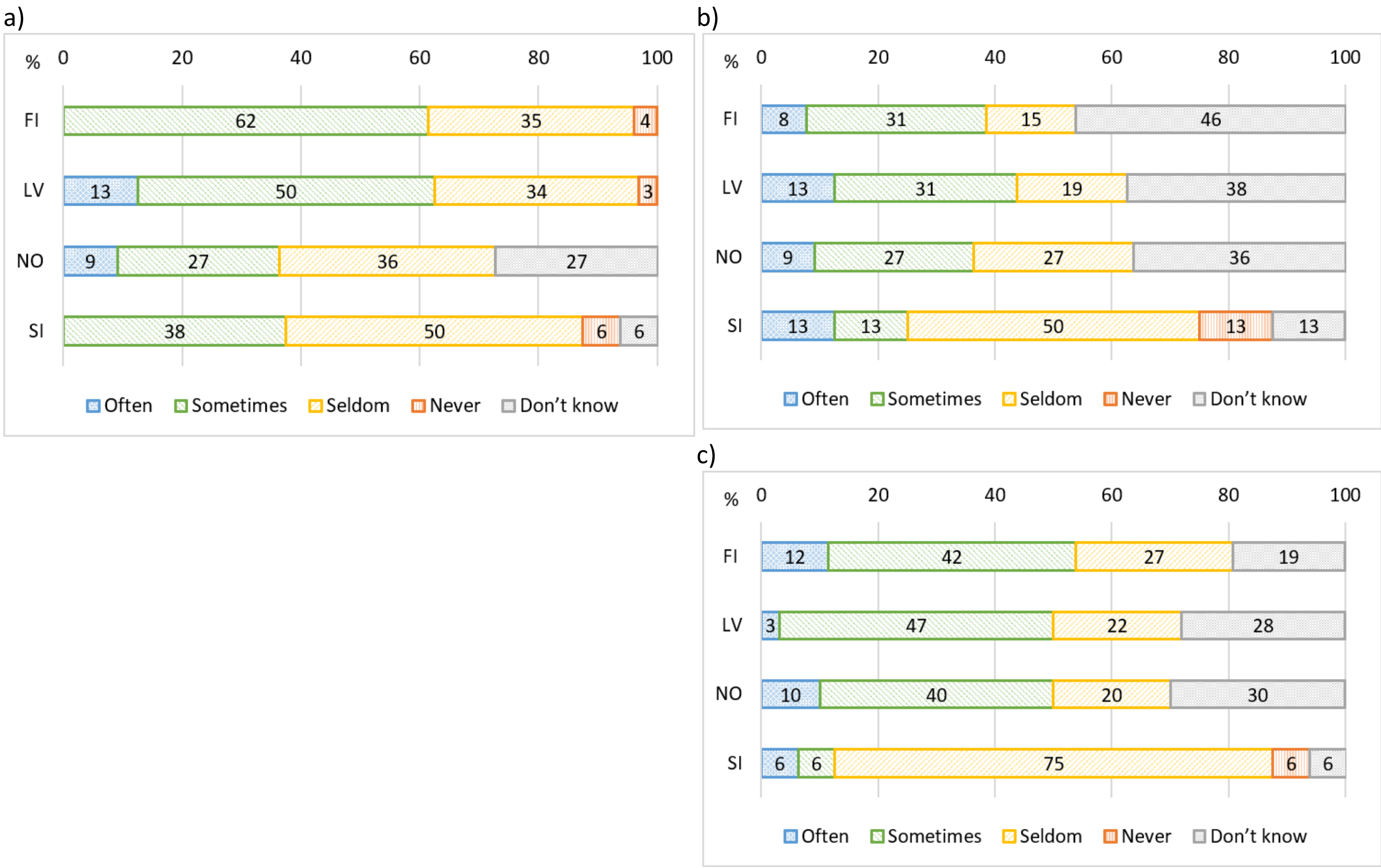

Figure S3. Identified challenges of workplaces in implementation of a) occupational exposure limit values (n=85), b) REACH authorisation requirements (n=85), and c) REACH restriction requirements (n=84), according to inspector survey responses from Finland (FI), Latvia (LV), Norway (NO) and Slovenia (SI).

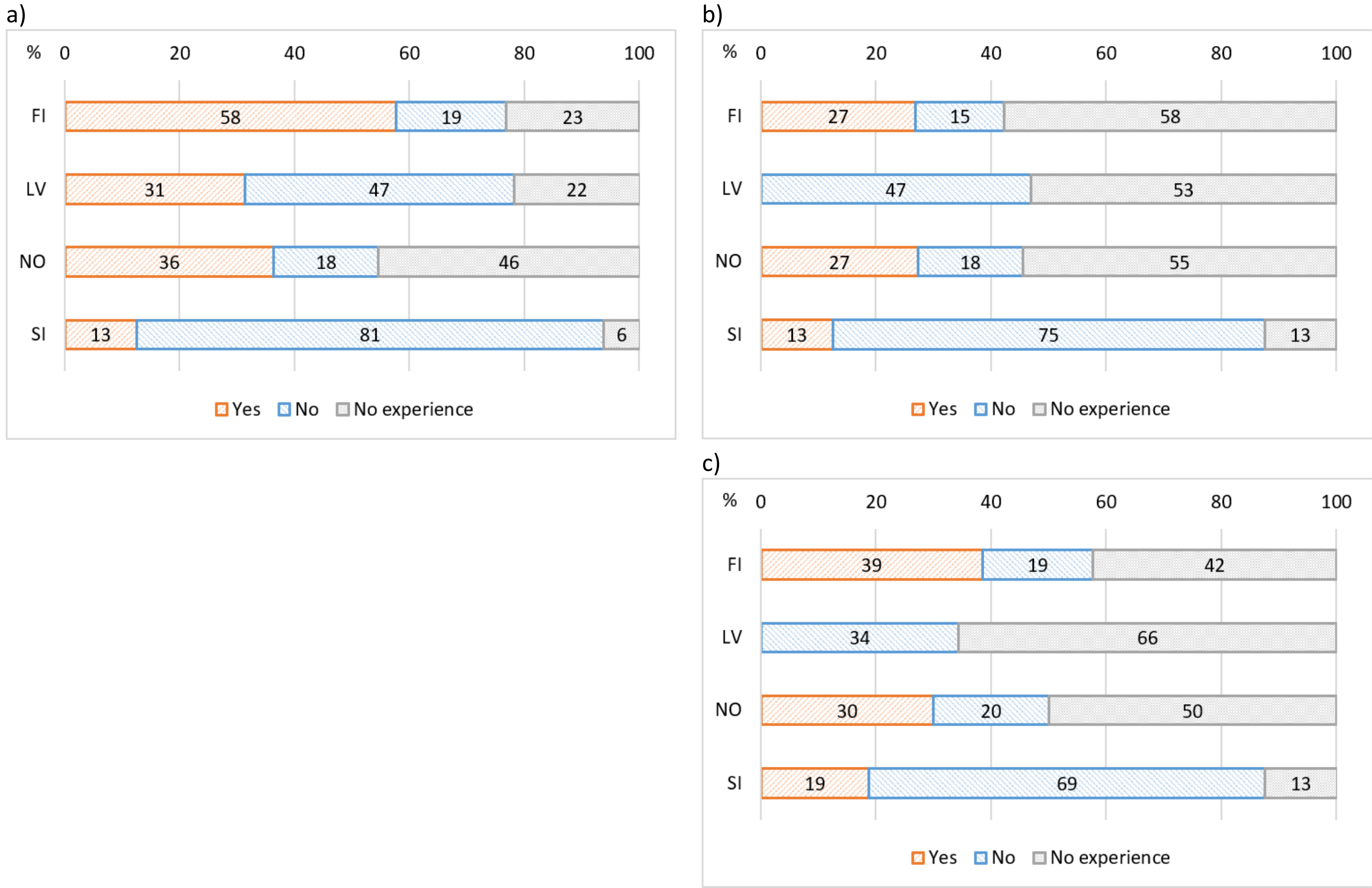

Table S1. Awareness of workplaces on OELs and REACH authorisation and restriction requirements, and ways to increase the awareness. Quotes from the labour inspector (I) and OSH expert (E) interviews from Finland (FI), Latvia (LV), Norway (NO) and Slovenia (SI).

Issues raised with representative quotes	Inspectors (by country)	OSH experts (by country)
Awareness on OELs and REACH requirements		
1. Wide variation in awareness between companies (size, sector, position in supply chain)	FI, LV, NO, SI	FI, LV, NO, SI
FI-I-01: In the big progressive workplaces where these things may have been considered, at least I personally go to fewer inspections when the assumption is, that things are in order there.<br>*LV-I-02: Companies for whom chemistry and chemical substances are not their core [business] activity but something is used occasionally, naturally lack awareness and knowledge because they do not perceive these risks as significant.*<br>*NO-I-03: Many of these companies [mechanical industry and car repair shop] are small and have little awareness of limit values.*<br>*SI-I-01: [Level of awareness] depends on the size of the organisation; some are more prone to safety measures, some less.*<br>*FI-E-08: ...if you do something that doesn't have much to do with chemicals, for which an adhesive or a solvent is only a small part of the whole, then they don't know that there are some special requirements or special restrictions for chemicals, authorisation procedures and so on.*<br>*LV-E-04: [Situation is better] in any industry where these chemicals are used, where there are very, very, very many of them.*<br>*NO-E-09: ...the size and type of company play a major role. [...] process industries are likely to be more familiar with this, as they tend to face greater challenges. On the other hand, it's also relevant for places like car repair shops, where there is often a significant lack of knowledge.*<br>*SI-E-02: The company in question [automotive service, international] is very motivated to perform significantly better than the legislative norms – they see this as a strong marketing point.*		
2. Availability and quality of expert services have an impact	FI, LV, NO, SI	FI, NO
FI-I-02: I think that occupational health care could also be a very strong support for this, so that when they carry out the workplace survey, they pay attention to the presence of these chemicals, chemical exposure agents in the workplace, which have a binding limit value, and how the workplace then ensures that the work there is carried out so that the exposure is below these binding limit values.<br>*LV-I-02: It depends on which occupational health and safety specialists are engaged by the company and how knowledgeable they are. How much attention do they pay to this risk? As a result, awareness in this area could be low.*<br>*NO-1-04: [...] so using their occupational health service properly is crucial when it comes to both understanding limit values and creating a solid risk assessment.*<br>*SI-I-02: OSH experts lack knowledge; some OSH experts do not know the basics of risk assessment. Also, the occupational healthcare system is poor.*<br>*FI-E-04: Workplace surveys [by occupational health care services] are of course one of them, where they are once again reviewed and yes, it is constantly on show in some way, to keep awareness up.*		

Issues raised with representative quotes	Inspectors (by country)	OSH experts (by country)
NO-E-09: You can get valuable support from a good occupational health service if you're interested in that kind of help. It's not always easy, of course, but a good occupational health service is a very important factor.		
4. Active participation in sector organizations increases awareness	-	FI, LV, NO
FI-E-08: All the companies that have the resources to participate in this work of the [chemical sector] association are reasonably well aware of what is happening on the occupational safety and health side or what is happening on the REACH side.<br>*LV-E-06: We have such a good thing as an [industry] association [...]. Then it's really easy, because [...] they know "the ins and outs" of the production itself, they know where to look. Not always at the country level, national level, this assistance [exists], but international.*<br>*NO-E-09: It's often through industry associations that we get the most up-to-date news. There are also newsletters from authorities, for example, or from selected consulting firms. [...] Public consultations are also an important way to stay informed about upcoming changes.*		
5. Awareness on OELs higher than on REACH requirements	NO	-
NO-I-04: We have had limit values [OELs] for a very long time, so most people know what they are—whether they relate to them or not. It is a value that is part of a risk assessment and can be measured.<br>*NO-I-03: Companies don't talk about authorisation and restrictions. They don't talk about REACH at all. It is completely unfamiliar to them.*		
6. Companies where adverse health effects occurred more aware	FI	-
FI-I-02: At some point, I have noticed that this awareness increases at the point when employees have some kind of health impact at the workplace.		
7. Companies with corporate responsibility programs more aware	SI	-
SI-I-03: Foreign companies are much more attentive to OELs as they want to be seen as responsible while Slovenian companies only deal with the problem when inspectors issue a warning.		
8. Companies/sectors with frequent inspections more aware	-	SI
SI-E-01: Some companies operations are more prone to frequent inspection audits, and in such organization, the driving factor for safety is fear from potential fines/penalties that can be issued by inspectors.		
Ways to improve awareness		
1. Training of OSH experts	LV, SI	-
LV-I-02: What could improve? What could enhance the knowledge and understanding of the specialists themselves on this issue? The occupational health and safety specialist is the one who notices and says, "This and that needs to be done, you need to know this and that, here is the information, let's learn, I will explain to you, and we will act correctly to protect ourselves".<br>*SI-I-01: Where there are outsourced OSH experts, there could be a problem with the experience or education; OSH experts can have a general education background and are not knowledgeable in chemistry.*		
2. Mandatory training requirements (diisocyanate restriction)	FI, NO	-

Issues raised with representative quotes	Inspectors (by country)	OSH experts (by country)
FI-I-01: The [diisocyanate] restriction, at least for now, increases awareness of allergenic/asthmagenic [substances] and increases safe working methods.		
NO-I-04: I think that restrictions leading to mandatory training make a big difference and are much better for these workers. But this only applies to one specific group of substances: diisocyanates.		
3. Campaigns and campaign materials	LV, NO	-
LV-I-01: Regarding awareness, I definitely believe that a campaign could be useful, perhaps with illustrative materials in the form of a booklet—structured in a way to promote understanding of what these matters are and what requirements need to be followed.		
NO-I-05: We have conducted several campaigns. We have published news updates whenever something new comes up, but companies need to actively participate in these activities, such as attending seminars we have invited them to.		
4. Targeted inspection activities	FI, LV	-
FI-I-02: Then we will focus our general surveillance more on some of these sites where such exposure agents occur.		
LV-I-02: I believe that our inspections, to some extent, draw specialists' attention to these issues. Then, hopefully, the level of awareness might increase, as the specialist also becomes interested and wants to learn what they should do, how to do it, and what the best practices are.		
5. Consultative attitude of labour inspectors	-	FI, LV, NO
FI-E-09: The open dialogue is really important, as if it would go like how I feel it goes in some countries, that there will be a punishment more or less immediately, then it won't promote that open dialogue, because then everything will be fine as long as they are not getting caught.		
LV-E-01: We've never ever had a labour inspectorate interested in this issue.		
NO-E-09: [...] They [inspection authority] should start with guidance and key topics, focused campaigns targeting industries, preferably in collaboration with relevant industry associations.		
6. Employers' commitment	-	FI, NO, SI
FI-E-07: In a way, it culminates in this managerial work and setting an example.		
NO-E-07: It might be more interesting for leaders to work with risk assessments if they understand that this is actually a management tool. [...] That they realize that HSE work and chemical safety are part of management, just like any other management task.		
SI-E-01: Depends on the viewpoints of the management of the company; some are more prone to safety measures, some less.		
7. Regular training	-	FI, LV, SI
FI-E-02: I would use energy to make sure that when people come to the workplace and come into contact with chemicals, we would use energy to assure internalization of these [chemical safety] things.		

Issues raised with representative quotes	Inspectors (by country)	OSH experts (by country)
LV-E-01: We have training every six months, and additionally, once a month, at team meetings, the occupational health and safety specialist reports on current events. *SI-O-02: Regular training of staff [is of importance].*		
8. Legal compliance services and tools	-	FI, LV
FI-E-03: We have our own service for monitoring legislation, where we also monitor changes in legislation.		
LV-E-06: We have even got our own software made internally where we ask for the updates into the search engine and automatically have a database of chemicals. […] We already have a software as a basis.		
9. Support from chemical suppliers	-	FI
FI-E-09: We want to take responsibility for that product as far as we can. It is good that we can perhaps get the level of awareness raised for those users who do not realize that they are chemical operators.		
10. Well-defined procurement practices	-	FI
FI-E-01: A delightful turn that has come to my ears is that procurement practices have started to be encouraged. When a new substance is being acquired, it is done centrally, and even occupational health care is asked for a statement.		
11. Better inclusion of chemical safety in vocational studies	-	FI
FI-E-02: The foundation should be laid at an earlier stage on how to raise awareness already when starting vocational studies. Readiness to understand these things as early as possible so that they don't come across as new and miraculous.		

Table S2. Impact of OELs and REACH authorisation and restriction requirements, and challenges hindering their impact. Quotes from the labour inspector (I) and OSH expert (E) interviews from Finland (FI), Latvia (LV), Norway (NO) and Slovenia (SI).

Issues raised with representative quotes	Inspectors (by country)	OSH experts (by country)
Impact of legislation		
1. OELs support exposure measurement and risk assessment	FI, NO	FI, NO
FI-I-01: For example, I would be more confident in obliging occupational hygiene measurements to be carried out when there is a binding limit value [OEL]. That the more of the letter of the law there is in the background, the [...] stricter obligations I would dare to give.<br>*NO-I-03: When it comes to limit values [OELs] - we talk a lot with companies about that because we issue orders for measurements, and those measurements must be assessed against the limit values, right?*<br>*FI-E-09: Somehow, the occupational safety and health limit values [OELs], as they are familiar for the people who directly consider those occupational safety and health issues, they have the way of thinking already there, in their spinal cords, how the occupational safety and health limit values are applied, where to find them, and what the hierarchy chain is, and how to reduce exposure.*<br>*NO-E-13: So where they use the limit values [OELs] to make measurements, it is indeed a very useful tool.*		
2. REACH requirements support substitution	FI, NO, SI	FI, NO
FI-I-02: The authorisation also had an impact, that process changes were made [...] or chemicals were replaced.		
NO-I-01: It is clear that when a substance becomes more or less banned and is practically withdrawn, they are no longer allowed to use it, which is quite an effective way to remove it. At least that puts an end to that exposure.<br>*SI-I-01: REACH is more about limiting of the use of substances – it is not referring to the process industry where OEL levels come more into focus.*<br>*FI-E-09: I feel that the authorisation procedure is so strict that if someone there is actually exposed to even a millimole, you won't get the authorisation. The idea is that the risks are under control, so I think it goes further.*<br>*NO-E-02: [...] if it comes from REACH or restrictions, it's usually a matter of "you don't use this," and whether there is a limit value or not doesn't really matter to us. For us, it's a bit black and white.*		
3. Restriction related mandatory training support occupational safety	NO	-
NO-I-04: I think that restrictions leading to mandatory training make a big difference and are much better for these workers. But this only applies to one specific group of substances-diisocyanates.		
4. OELs support inspectors in their work, REACH requirements less so	FI, NO	-
FI-I-01: I haven't come across these workplaces [with REACH requirements] in my career as an inspector, it is more about OSH legislation, I play with.<br>*NO-I-01: When you have an exposure limit [OEL], you can argue why it is necessary to measure and why it is necessary to reduce exposure, at least below the limit, and preferably even lower. It is definitely an important tool.*		

Issues raised with representative quotes	Inspectors (by country)	OSH experts (by country)
5. REACH requirements may hamper European businesses	-	FI
FI-E-09: If we stop doing a process and there is no substitute, then it will be done somewhere else. The number of people who are actually exposed will not decrease, but the business and jobs will disappear from Finland.		
Hindrances of impact		
1. Lack of competence/expertise	FI, LV, NO, SI	NO, SI
FI-I-01: I would say that it is not well known as we are still wrestling with if you have safety data sheets, and do you know that a risk assessment should be done. This goes much further and deeper.<br>*LV-I-04: Unfortunately, some employers have a very weak understanding, so to speak. Occupational health and safety specialists often say that their first battle is with the employer, to explain why something is necessary. Simply pointing out the existence of regulatory requirements that must be followed, unfortunately, does not always get through to employers.*<br>*NO-I-02: I would be very surprised if I walked into a small company, like a workshop, and found that they were aware of a new exposure limit for a welding contaminant or a type of solvent - because they have barely even read a safety data sheet.*<br>*SI-I-1: On many occasions, there are OSH experts hired, who only have general knowledge, who may not be competent in chemistry or hazardous substances. The problem is also that the company management lacks detailed knowledge and believe what they are told by not very competent OSH "experts".*<br>*NO-E-10: Another barrier is simply competence. The knowledge of REACH, exposure, and occupational hygiene is generally low. [...] The general knowledge at all levels, including among us experts, and the number of experts available, is insufficient.*<br>*SI-E-03: The knowledge of hired consultants is usually very low.*		
2. Lack of financial resources	FI	FI, LV, NO, SI
FI-I-01: Money is one thing that matters quite a lot, or at least it slows down the taking of measures.		
FI-E-07: Convincing that this [improvements in exposure control] really must be done, is not always easy if there is no clearly demonstrable repayment period.<br>*LV-E-04: To my mind [...] the impediment, probably always, is the budget, or funding, that is available.*		
NO-E-12: I have noted one thing as a challenge, regarding implementation, and that is the economy of the company.		
SI-E-01: The rate of implementation of new safety measures is related to associated costs – if there are high direct and indirect costs related to new safety measures, the reluctance is higher, and the implementation rate usually less successful.		
3. Complexity of legislation	LV, NO	FI
NO-I-04: But we do have chemical regulations, particularly REACH and CLP, which are too complex. [...] They are extremely complicated, difficult to navigate, and linguistically challenging to understand, making them a major challenge for companies.<br>*LV-I-05: Yes, I would also like complex matters to be presented in a simplified way, making them easy for an average person to understand.*		

Issues raised with representative quotes	Inspectors (by country)	OSH experts (by country)
FI-E-06: Yes, it's not always the easiest to read, either the ones that you start to find out on the internet yourself and end up looking at ECHA or all the REACH stuff, so you have to concentrate on the reading comprehension and wonder what this really means.		
4. Poor information flow in supply chains	NO	-
NO-I-05: They have seen that the substance is subject to authorisation, but since they have had no communication with the supplier, they know almost nothing about the conditions that apply to its use.		
5. Technical challenges in reaching the limit values	-	FI, NO
FI-E-08: In some of those limit values, the concentrations are already so low that it is not technically possible to achieve such good filtration or something else, then the only option is to use the mask.<br>*NO-E-04: [...] or, for example, the use of masks in combination with safety glasses – we have experienced that some types start to fog up if people are working intensively, often in the smelting industry at high temperatures, which then results in reduced visibility.*		
6. Chemical safety software do not consider REACH/OELs	-	FI, NO
FI-E-06: In some cases, our risk assessment may be marked in our software in a way that it should be re-examined, but this [changes in legislation] does not affect it, for example. It does not consider limit values, let alone other restrictions and REACH requirements.<br>*NO-E-07: Most of the chemical inventory solutions available on the market today, I believe I can say, do not take limit values into proper account. Instead, risk assessments are made based on the knowledge one may have at hand.*		
7. Time-consuming processes	-	NO
NO-E-09: It's a challenge that it takes a long time for a company to adjust. When a new limit value is introduced, just the process of measuring often takes more than a year. And then you also have to implement measures on top of that.		
8. Poor safety attitude	-	SI
SI-E-01: Attention not given to these issues until inspectors issue a warning		
9. Restrictive business practices hinder cooperation	-	FI
FI-E-09: [Earlier] when competition law gave us the opportunity to do these kinds of safety walks, or to visit the premises of other companies to see how things were arranged, at least these [safety management] models were easily copied.		

Table S3. Use of exposure measurements and limit values. Quotes from the labour inspector (I) and OSH expert (E) interviews from Finland (FI), Latvia (LV), Norway (NO) and Slovenia (SI).

**Issues raised with representative quotes**	**Inspectors (by country)**	**OSH experts (by country)**
Requiring exposure measurements (inspectors)		
1. Measurements required when high concentrations expected	FI, LV	-
FI-I-02: If the inspector has been able to say that there have been reports of occupational diseases here and the employees during the inspection then complain that the concentration in the air here is so horrible that it stings the eyes, […], so at that point the inspector has obliged [measuring].<br>*LV-I-01: When it is obvious that something is too much, as it should be […], we recommend that these measurements be taken.*		
2. Measurements required after process changes	NO	-
NO-I-03: If there have been significant changes in production, we can require new measurements even if less than three years have passed.		
3. Measurements seldomly required		
SI-I-02: It is difficult to have the accredited laboratory at one's disposal constantly, so it is used only occasionally or periodically.	SI	-
Conducting exposure measurements (workplaces)		
1. Measurements conducted regularly	-	FI, LV, NO
FI-E-07: Of course, we [do] occupational hygiene measurements to ensure that the concentrations are not exceeded, or the limit values are not exceeded. Of course, these are considered in the risk assessment.<br>*LV-E-06: Another criterion that is taken into account is the Cabinet of Ministers regulations, which specify how often, when, and with what frequency*.<br>*NO-E-01: We carry out regular measurements on what we consider to be relevant.*		
2. New limit values trigger measurements	-	FI, NO
FI-E-03: When those new limit values are set and binding limit values are set and updated, we monitor them and then react accordingly.<br>*NO-E-01: If there are any changes in processes or limit values, we do new measurements or at least assessments*.		
3. Changes in work processes trigger measurements	-	LV, NO
LV-E-06: That [introduction of a new chemical or increase in production volume] is a change in the work process, and then we measure.<br>*NO-E-01: If there are any changes in processes or limit values, we do new measurements or at least assessments*.		
4. Recommendations from occupational health care trigger measurements	-	FI

Issues raised with representative quotes	Inspectors (by country)	OSH experts (by country)
FI-E-01: [Occupational hygiene measurements] have been done because occupational health care has recommended them. This was the joy of the week; that's how it should be. The vibes have been that this is not how it goes.		
5. Complaints from employees trigger measurements	-	LV
LV-E-02: Laboratory tests were ordered, but they were more related to complaints from the employees.		
6. Measurements conducted if required by authority	-	NO
NO-E-07: I believe that exposure measurements are mostly carried out as a reactive measure, meaning in response to a requirement.		
7. Exposure monitoring replaced by medical examinations	-	SI
SI-E-02: Occupational health yearly check-ups have taken over the role of monitoring.		
Choice of reference values		
1. OELs preferred over DNELs	-	FI, LV, NO
FI-E-03: [DNELs are] probably not used as actual reference values yet or measured and reported that now they have been compared to the DNEL value. Maybe there hasn't been a need yet.<br>*LV-E-06: But we do not use them [DNELs] further, for example, we refer everywhere to OEL, and we don't write that OEL is like that, but DNEL is like that.*<br>*NO-E-01: We use limit values [OELs].*		
2. Most stringent limit value chosen	NO	FI, NO
NO-I-05: What we have seen is that when DNEL values are lower than exposure limits [OELs], one would expect compliance with the lowest value.<br>*FI-E-09: [...] in any case, legally you have to follow the stricter value, that no alternatives are seen there.*		
NO-E-01: We would have used the strictest limit value, or the strictest value to be absolutely sure.		
Sufficiently low exposure levels		
1. Measurement results just below the limit values accepted	FI, LV, NO	SI
FI-I-01: In the eyes of the law, it [OEL] is an absolute numerical value, and it is ninety-nine percent of the value, so no matter how hard you try to explain whether you know statistical mathematics, whether you know how to calculate an error, it doesn't really sink in.<br>*LV-I-02: Adequate is what does not exceed those [occupational exposure] limit values.*		
NO-I-02: If we believe they are below the exposure limit [...], we consider that to be good, as long as they do not make changes to production conditions and so on.<br>*SI-E-01: Basic understanding is that if we are within the legal limits, the safety is sufficiently addressed.*		
2. Measurement standards (e.g., EN 689) used to address uncertainties	NO	FI, NO, LV

Issues raised with representative quotes	Inspectors (by country)	OSH experts (by country)
NO-I-03: We have guidance available on our advisory pages, which is based on the standard- is it called 486? - that covers this topic. If you have conducted […] measurements, you compare them with […] % of the exposure limit […]. That is what we adhere to.<br>*FI-E-08: There is a direct evaluation of how far it is from the limit value [in EN 689], so then measures are taken or recommendations for the use of protective equipment.*<br>*LV-E-01: We rely on the competence of the occupational disease laboratory specialists at Riga Stradiņš University, who carry out the laboratory tests for us [the laboratory follows measurement and laboratory standards].*<br>*NO-E-01: We are following the new guidelines […]. Now it's about 20% of the limit value. If it's below 20%, then it's fine. It really depends on how many samples we take, but generally, we take 6 measurements per group. And then we are within the limit. We need to be well below it to not follow up.*		
